# Questions for the Psychology of the Artful Mind

**DOI:** 10.3390/vision3040067

**Published:** 2019-11-21

**Authors:** Carmelo Calì

**Affiliations:** Department of Humanities, Università degli Studi di Palermo, 90133 Palermo, Italy; carmelo.cali@unipa.it

**Keywords:** perception, psychology of art, visual art, paleoanthropology, cognition

## Abstract

This paper reconstructs the “Arnheim’s puzzle” over the psychology of art. It is argued that the long-established psychological theories of art do not account properly for the observable variability of art, which provide the phenomena of interest whose psychological factors need to be discovered. The general purpose principles of such theories, the ensuing selective sample of art phenomena, and assumption of conventional properties of aesthetic experience make the predictions and the findings of the theories unrepresentative of art. From the discussion of examples drawn from contemporary visual arts and the presentation of the debate on the emergence of the cognitive capacities of art in paleoanthropology, a construct is presented on the specificity of the cognitive capacities of art and its anchoring to perception, which solves the puzzle and has implications for research and teaching psychology of art.

## 1. Introduction

Arnheim [1] submitted that art production and experience are as subject to psychology as any other form of cognition and that the study of mind would need the psychology of art. However, the latter was hardly providing groundbreaking results by comparison to general psychology. Arnheim claimed that the psychology of art failed to keep pace with other areas of psychology because the qualitative description of phenomena was disregarded by the quantitative methods of psychology and because there was no direct contact between psychology and art. Notwithstanding, any domain of psychological research could be found in art, the psychology of art was missing the chance of providing aesthetics with sound facts. Nowadays the strict divide of measurement under controlled conditions and generalization from the observation of well established facts and founded concepts, which Arnheim drew with disappointment, is no longer meaningful. A wealth of evidence has been gathered on cognition and many attempts have been made to account for visual arts and music according to the concepts and the models of the Cognitive Sciences [2,3,4,5,6], not to mention the great number of papers published in journals dedicated to the empirical study of art and aesthetics. Yet Arnheim’s claim seems to be still sound to a certain extent. No one has reason to complain about the scarcity of relevant studies on art any longer as Arnheim did, but art seems still to elude a thorough psychological explanation. Studies in psychology of art often (1) pick one model of a cognitive function among those built in a specific field of research, (2) apply it to a selected class of works, (3) emphasize an overarching characteristic of cognition that (3.1) cuts cross-wise artistic and ordinary cognition or (3.2) is a rather recent construct, like creativity that is an artistic value only since the Avant-Garde, or (4) extend art cognition to aesthetic experience. One can dub, then, this situation as the “Arnheim’s puzzle” over the psychology of art: the firm scientific basis and the interest in artworks of various styles, genre, and age are not sufficient for it to give a comprehensive account of art and cognition. There are implications for teaching psychology of art as well. Which kind of scientific evidence can one present and at which degree of specificity in connection to art? Does the discipline have a proper subject-matter even though it cannot but refer to theories and evidence from other disciplines? Can one set a standard for presenting the value of alternative theories or contrasting evidence?

## 2. Theoretical Reductionism

Teaching issues are related to research issues. Berlyne [7] acknowledged that the phenomena of the experience of art are “the most complex” with which the behavioral sciences have to deal. The aesthetics “from below” of Fechner [8], who measured the correlation between formal elements and preference, was early recognized as an oversimplification for the problems of the psychology of art. Three main strategies were developed to deal with that complexity. 

The New Experimental Aesthetics traced the cause of art to the behavioral constant of diversive exploration in search of pleasurable stimuli, which provide an optimal balance between the inflow and the variability of information. The experience of art is a highly elaborated form of exploration. Artworks are patterns characterized by “collative” properties, namely properties that arise when beholders compare their basic features, which take on optimal values along biologically significant axes. They impart enjoyment to the beholder as a pay-off for her effort into that complex modality of exploration. The psychological account of art involves psychophysical measures of physiological indicators of the arousal level associated to the pleasure induced by stimuli, behavioral measures of visual or auditory exploration, estimates of pleasure and preference rankings through survey scales, and the statistical analysis of the collative properties of stimuli [9]. 

The Gestalt Psychology considered artworks as “pure” instances of the law of self-organization of psychological and physical systems, which implies a tendency to equilibrium. Equilibrium is the end state at which the minimum principle, for which a system tends to simplicity, is balanced with the external constraints that force it to maintain an articulation, the most regular at the prevailing conditions (the Prägnanz principle). Self-organization is carried out in ordinary perception and in the production and the perception of artworks through the interaction of grouping factors. In the works of visual art and music artists employ the factors to pit perceptual properties of form, color, spatial layout or sounds against one another on the plane of the picture, the volumes of solids, the simultaneous or successive stream of notes. If clear-cut tensions between perceptual simplicity and articulation ensue, the pattern conveys a meaning that can be perceived by the beholder [10,11]. The psychological account of art involves the description of the perceptual organization of the artworks and the study of its systematic correlation with observers’ reports and the experimental evidence on perception. 

The Cognitive Psychology tried to find the explanation of art by building models of the processes and the structures underlying the manipulation of representations. Representations are ordinarily manipulated to collect and process information from the environment, according to rules or schemes that because of capacity limitation enable the anticipation and control the selection of the available information. Artworks are patterns of symbols that are material representations in visual or auditory format. Artists produce such patterns by working on a “medium”, that is the chosen material means and technique, to yield a new source of information in the environment. The pictorial or the musical medium is a communication vehicle between artists and beholders. The material representations are equivalent to the mental representations artists intend to communicate to beholders. Therefore, art production requires the artists to master a symbol system and to possess the procedural knowledge (motor and technical skills) to manipulate the medium. Indeed, the properties of material means are processed through the chosen technique to the finest details to convey, expressively, the meaning of the symbols. Beholders for their part must learn the symbol system to construe the symbols and to reconstruct the intended representations. The production and the experience of art amount to an intelligent functioning mode of the mind [12,13,14]. The psychological account of art involves the abstraction of the variables from the symbolic pattern of artworks to discover the representational rules with which artists and beholders comply, or the selection of the parameters for models which simulate the judgments of beholders, the evaluations of art critics, and the principled claims of artists. These three strategies treat art as the class of highly complex phenomena with many dimensions. As Crozier and Chapman [15] have early recognized, however, the New Experimental Aesthetics and the Gestalt Psychology get down to examining artworks from the standpoint of general principles on cognition, which are independent of arts. Since the expectation was that their effects should be present in the artworks or in the stimuli contrived to study the relevant dimensions of artworks in experimental conditions, the research carried out within those theoretical frameworks aimed at measures or observations of the features of experience which matched those effects. Eventually, the systematic correlation between the independent variables or the descriptive units and the features selected on the basis of theoretical expectations was considered a confirmation of the theory. If the development of the Cognitive Sciences and the manifold examples of artworks which do not fit the symbolic paradigm are considered, this argument holds also for the Cognitive Psychology of art. It is noteworthy that from the evidence to which the three strategies appeal one could tell more about the theories than about phenomena. Some conclusions made a contribution to understanding art, but only with regard to the aesthetic experience of a restricted sample of stimuli and artworks. These theories have reduced the subject matter of the psychology of art to the domain of application of theoretical principles, beyond which they are likely to prove to be not specific to the explanation of art. Consequently, it is not clear whether measures or observations can decide between alternative theories. What the theories missed to account for is indeed the variety of art. 

## 3. Art as a Source of Variability

Arnheim [1] had argued that psychologists tend to cling to conventional notions about art instead of taking up the challenge issued by the study of the production and perception of artworks. Indeed, artworks vary in manifold measures and respects, hence the properties they display for the beholder to grasp their meaning and to ascribe artistic value to them often defy the explanatory constructs of theories. For instance, what are the implications of the neurobiological basis of beauty [16,17] for the experience of art in the presence of Goya’s *Saturn devouring one of his sons* (1821/1823)? If pleasure is taken as explanatory variable of art, which role does it play in the experience or in the attribution of value for a painting like De Ribera’s *Saint Onophrius* (1630), in which the viscosity of oils exhibits the decay of bodily tissues? Pleasure or hedonic value can be considered to arise from a general purpose reward system, which is modulated by a specific behavior in search for stimuli with particular characteristics or is specialized for making appraisal of beauty as emergent property [18,19,20]. However, it seems to be irrelevant to the experience of such artworks as De Ribera’s whose technique and represented subjects purposefully rules it out, even if pleasure is construed as the hedonic value that is the net effect of antagonistic reward and aversion systems [7]. Like many instances of modern art, albeit differently, the pictorial and representational content of De Ribera’s painting have been realized to bring about the perception of something predominantly unpleasant. Even if the enjoyment of artworks is associated to the enhancement of the attractiveness or pleasantness of a stimulus due to the exposition to or the familiarity with it [21], the question is not settled. The aesthetic experience one has the first time she is presented with Oppenheim’s *Le déjeneur en fourrure* (1936) would still provide a counter-example. Indeed, this genre of artworks aim at appearing highly discriminable for beholders to appreciate the inconsistency of common sense concepts of things and actions the work of art displays. 

There are in fact models that may justify the use of conventional notions like beauty or pleasure even for experiences of artworks which elude them by design. Cupchik [22] submits that the features of artworks are affectively coded and projected to arousal and pleasure levels only once an aesthetic mode of interaction with what they present is activated instead of a pragmatic mode. The aesthetic mode sets a cognitive distance from the work of art. The greater the distance, the more the pleasure is felt even for the presentation of something negative. However, artists who belonged to the Cabaret Voltaire or the Dada movement prompted those who attended their happenings or made experience of their works to be committed to the criticism of artistic common sense. The latter were and still are aware of the context in which such artistic expressions were framed and recruit only some of their cognitive resources for the aesthetic experience, which are then detached from usual activities. Nonetheless the greater the amount of cognitive resources invested within the particular frame of such art experience, the more they understand and appreciate the intended meaning.

Art is characterized by huge variability, which should not be neglected by psychological theories of art in the name of general principles independently assumed. Massironi [23] argued that psychology should consider art as a problem, rather than as something given on the basis of the assumptions about what art should be, which derive from the belief that the independent general principles are true. Psychology should therefore consider artworks as the distinctive phenomena of a scientific domain that needs to be investigated in its own right. 

One may refer to the history of art to identify the dimensions of variability the domain should have. The history of art regards 

(a.1) artists as the bearers of iconographic and stylistic change, 

(b.1) works under the respect of established or new stylistic types within well-defined genres, 

(c.1) experienced or naive beholders’ judgments on the value of artworks insofar as the latter embed canonical or groundbreaking representational conventions. 

The corresponding dimensions of the psychological domain, which capture art’s variability can be phrased as follows: 

(a.2) the capacity of artists to turn perceptual into representative features so that beholders can recognize something in what artworks present; 

(b.2) the ability of artists to master the techniques to solve the problems that the medium poses for the representation, in that the material properties of the surface or the volume of the medium must be replaced by those of the representation and, consequently, the former act as constraints on (a.2); (b.3) the capacity of beholders to understand the representation and its “mode of appearance”, namely the represented scene regarded under the respect of how it has been obtained through the alteration of the medium given the constraints and ability of (b.2). 

The cognitive capacities are the psychological factors of the observed variability of art. They build a sort of perceptual knowledge shared by artists, beholders, and critics or historians, on whose grounds artists display their skills and observers assess the value of the artwork and enjoy the experience of it accordingly. It is reasonable to submit that this knowledge is anchored to perception because the capacities involve exploiting the regularities of perception to construct the representation, to explore the properties of the surface or the volume of the medium, to ascertain the consequence of their alteration through the chosen techniques, and to anticipate the mode in which the artwork will appear [24,25]. 

Contemporary visual arts provide manifold examples. Brightness and color contrasts are found in Seaurat’s *Nudes* series, Signac’s *L’orage* (1895), Cezanne’s *Mont Saint Victoire* (1904/1906). These features of color perception are variously employed to give a structure to the depicted scene and at the same time to display the peculiar control of the variability of the kind of the chosen colors. Perceptual features have been used also by Avant-Garde movements to extend the scope of the representational features of art, hence, to dictate a radical change of how the artwork appears and art making is conceived. In the *Manifesto of Futurism* Marinetti claimed that the world of art would be enriched by the beauty of speed, “a new kind of beauty”. Features of the perception of movement became soon eligible for the set of pictorial and plastic properties in Balla’s *Dynamism of a dog on a leash* (1912) or Boccioni’s *Unique Forms of Continuity in Space* (1913). Duchamp employed clues to perceived motion both to give a structure and a subject to his *Nu descendant un escalier* (1912), and in 1935 he constructed his “rotoreliefs” with depth from motion as perceptual material. Later Tinguely and Calder will present concrete machines whose moving parts and mechanisms test the perception of movement of the beholder. 

In the *Technical Manifesto for the Futurist Sculpture* (1912) Boccioni advocated the use of different material for the plastic arts up to conceiving of a single sculpture made of 20 heterogeneous materials like glass, wood, cement, cardboard, horse hair, leather, clothes, mirrors, and electric lights. In this case the visual textures of materials are conceived as a perceptual means that brings about a radical change of the concept of the artwork as homogeneous unity. 

In contemporary art the introduction of perceptual properties to be turned into new representational features increases the variability of art. The problems that media pose to artists vary accordingly and require that artists would use their technical abilities to find solutions that may end up eluding long-established concepts of art and of aesthetic experience. However, artists expect beholders to understand the representation and its mode of appearance. This expectation is as much anchored to the shared perceptual knowledge as is the capacity of beholders to find traces of the abilities of artists to alter the surfaces and the volumes of the medium to provide new solutions to the problems of representation. On that basis, beholders attribute or deny artistic value to the work. 

It is noteworthy that this increase of variability is found also in contemporary music and Tenney [26] discusses the implications for the theory and the experimental psychology of music. Indeed, the wide range of variability of art is a challenge for the psychology of art. Instead of deriving from the outset the properties that aesthetic experience should have on the basis of general principles on the mind or on the art, a psychological account of art should

(1) make an hypothesis on the specific contribution of cognitive capacities to this variability,
(2) collect as many representative samples of artworks as possible,
(3) define constructs and variables to test their validity as a function of the amount of variability they capture over the dimensions of interest.

## 4. Some Issues in the Cognitive Paleoanthropology of Art

Interesting clues to the specific contribution of cognitive capacities to art may come out of the debate on the emergence of those capacities, from which derive the “cognitive markers” that qualify fossil records and prehistoric findings as antecedent or instances of art. The cognitive markers are the ability to act beyond the immediate spatial and temporal localization (abstract thinking), the strategic ability, the ability to innovate behavior, economy and technology, and the ability to represent something with visual or vocal signs. Consensus has been reached on the fact that these markers came to be expressed by cognitive functions realized in the context of different activities like resource acquisition, stone tools manufacturing, and the anticipation of what the use of tools requires. Standard accounts have defined the cognitive antecedents of those capacities and their evolutive time scale. They could have developed from the socio-cognitive abilities needed to communicate and to build groups. Body decorations, jewelry and beads made as sign of belongingness to a particular group may have acted as an earlier selective stage for the production of art. Alternatively, they could have developed from the need of expression dictated by mate selection, which triggered the habit of symbolic representation. The elaborate handles of the hand axes from Western Europe in the Upper Paleolithic exhibit strength, cleverness, intelligence, creativity in the same sense in which colors, shapes and rituals signal gene fitness in the competition that takes place in socially layered early human groups. The period in which the capacities of art could have emerged is the “Transition period” (45,000–35,000 years ago) characterized by the Aurignacian technology. In this period, indeed, the archeological records show a sudden change. Humans were building objects, like fine hand tools with decorative handles, which wholly or in part were not meant to realize a function and were made of new materials like flint, bones, antlers, ivory, and stones. Miller and Lewis-Williams claimed that the first instances of figurative art like markings, pictures, statuettes appeared just in this period [27,28]. 

However, the long wooden spears discovered in the Schöningen site, whose skillful shaping required the technological know-how that qualifies as antecedent of artistic manipulation, are dated back to 400,000 years ago [29]. Strong evidence against the standard account is provided by the findings of pigments processed by means of grindstones and of ochre production toolkits together with red or yellow tinged pieces of rock, which were engraved with abstract patterns and used to decorate the body and clothes and to produce pigments through scraping, because they are dated to respectively 250,000 and 100,000–70,000 years ago [30]. In those periods, archaic hominids like the Neanderthals were populating Africa and Europe. They coexisted with anatomic modern humans for 15,000–10,000 years. It has been argued that late Neanderthals were behaviorally modern [31,32]. Records from the site of Skhul and Qafze show that Neanderthals settled in that region of Israel 60,000 years ago after the colonization of early modern humans. It took modern humans 10,000 years to regain the territory, which attests Neanderthals capacity to adapt and to exploit one’s demographic advantage. Regarding the cognitive markers of art there are also significant findings. Flutes made out of animal bones have been found in France, Slovenia, and Germany and dated back to 53,000–40,000 years ago [33,34]. Finally, a depiction of red linear motifs, a red painting of speleothems, and a hand stencil in Spanish caves was dated at least to 64,000 years ago [35].

Such findings have important implications. Is biological modernity a necessary and a sufficient condition for cultural modernity? It is reasonable that it is the case. However, how can one account for evidence of Neanderthals’ artistic behavior? The claim of behavioral modernity of Neanderthals sounds logically inconsistent, but the fact that modern humans outcompeted archaic humans cannot be explained by reducing cultural modernity to anatomical modernity. Neanderthals most likely borrowed the symbolic or artistic production by anatomically modern humans [36]. Therefore, the evidence about their coexistence would suggest that the geographical, the behavioral, and the cognitive contact acted via competition as the catalyst of cultural complexity and technological innovation [37]. 

One can submit, then, that a common psychological factor has fostered the connection of cognitive capacities, which were developed independently in different contexts of activity, in the composite capacity of art. This should be a “minimal” factor, because it could have been shared by such different kinds of hominids. The least common factor may have been the ability to solve problems in natural and social environments by exploiting and manipulating the mode of appearances of that which characterized the environment shared by those different kinds of hominids. Perception of animals in their surroundings and of the effectiveness of camouflage may have been the antecedent to which the capacity of tracing marks and drawing forms for body decoration and ornamentation is anchored. The use of symbols to convey information on social rank may have been the antecedent to which the perception of the skills and abilities needed to make them is anchored. Such skills and abilities may have been later displayed in a different form of communication, that is making marks on surfaces and modeling volumes. 

Since cultural modernity eventually led to art, paleoanthropology indicates that distinct capacities anchored to basic modules of perception, like those above mentioned, may become specific to art. In ordinary perception of the environment such basic modules serve the capacities of picking up regularities and solving problems like those posed by camouflage and signaling or recognizing social ranks. If those capacities are transferred to the restricted domain of producing artifacts, the underlying modules become connected to one another and turned into the abilities of making form, colors, and contours appear in surfaces and volumes in a such a way that someone else can recognize the skills displayed by this modification of surfaces and volumes.

## 5. Settling Arnheim’s Puzzle

From the debate on the archeological records, the inference can be made that the capacities labelled as (a.2), (b.2), (c.2) give the specific contribution to art because the perceptual modules they imply subserve the aim of making a representation perceivable in surfaces and volumes, by altering the perception one would have of them in ordinary experience, so that who perceives the representation recognizes its mode of appearance as meaningful as its subject matter. The connection of abilities involved by (a.2), (b.2), (c.2) enable artists to solve the problems posed by the alteration of surfaces and volumes as well as beholders to appreciate the mode of appearance and to derive the mastery of artists from the perceivable traces of the alteration of the medium. Artworks are not deceptive. Their experience depends as much on recognizing the representation and its aspects as on perceiving the traces of the alteration of surfaces and volumes by means of the chosen technique. In this sense the perception of some property of the medium or of the effects brought about in it is preserved [24].

The construct of “composition” can be introduced to denote the representation and its mode of appearance, but always related to the perception of the medium preserved through transformations. The composition consists of the arrangement of boundaries, shapes, colors, textures, and motion clues, through which beholders recognize something else from the medium, namely a represented figurative scene or an abstract pattern. As this arrangement derives from the alteration of the medium, that is to say the manipulation or transformation of the way surfaces and volumes would be perceived in ordinary perception, the composition includes also the mode of appearance of the representation as a salient feature. The mode of appearance is perceived as the effect of one or more transformations operated on its surfaces and volumes. For an artifact to be valued as art, indeed, the requirement holds that the beholder can recognize anything in, over, under, in front, or beyond the medium, but always distinct from it, as well as reconstruct the abilities of the maker. This holds regardless of the fact that the artistic value is attributed, rejected or even withdrawn at the time in which it has been made or only afterwards. Be that as it may, the representational shapes, colors, textures, movement, and motion clues are obtained by the alteration of the medium, which can go from erasing to exploiting in full medium properties. *Trompe l’oeil* paintings and frescos or Canova’s polished sculptures are example of the first case. Training at the Bauhaus with the treatment of surfaces of various materials by means of different tools to try out their visual and tactile properties as well as their translation across different perceptual modalities falls within the second case [38,39].

The term “composition” is preferable to “representation”. The latter does not imply the reference to the perceivable array of the traces of medium’s alteration, which plays a role in appreciating the mode of appearance and the artist’s abilities. “Composition” denotes a composite phenomenon and allows to take into account the difference between the representation, its mode of appearance, and the properties of the surface and the volumes of the medium. The perception of this difference, which may be controlled by artists, is one of the determinants of the experience of art. It may vary and the extent to which it does contributes to the variability of art. At one end, the difference may approximate the null value for the composition like, for instance, in Burri’s *Wood Sp* (1958) and *Combustion* (1961). At the other end, it may approximate the null value for the alteration of the medium like in Duchamp’s ready-mades, when the artwork consists of changing the context or the usual frame of reference where one usually makes experience of the object, and in Kosuth’s *Three Chairs* (1965), where the emphasis is on the definition rather than objects. In-between cases are those in which the medium is altered in a way that appears inconsistent with the composition, like in Oppenheim’s *Le déjeneur en fourrure* (1936), or that occludes the composition like in Man Ray’s *L’enigme de Isidore Ducasse* (1925).

The cognitive capacities (a.2), (b.2), (c.2) are independent from one another because the perceptual modules involved by each of them are ordinarily applied to distinct parts of the environment to pick up different kinds of information or to plan actions. The domain of the psychology of art is set by the way such modules become connected and specialized for an aim different from that pursued by ordinary perception. Issues for the psychology of art are (1) the specialization of perception, which is disengaged from understanding the environment and recruited to turn perceptual features into the elements of representational arrangement, (2) the specification of the mode of appearance of representation as effect of the alteration of surfaces and volumes, (3) the difference between the perceiving the composition or the properties of the medium.

The specialization means the restriction of cognitive abilities to the solution of the problems of representation from the artist’s standpoint. Artists select perceptual chunks of the environment, like discontinuities of surfaces or transparency, turn them into representational elements through the alteration of the medium ’clipping’ them together in a representational arrangement according to a peculiar style. Alternatively, artists select some perceptual properties of the medium and emphasize them through the chosen technique to present the surface and the volume as the subject of the work. The specialization holds also for the problems of recognition and evaluation from the standpoint of beholders. 

The psychology of art needs not a special method. Any question about making and experiencing art can be studied like in standard Cognitive Science by means of constructing conceptual definitions, building models of phenomena, testing the hypotheses, or the predictions of models. Due to the perceptual anchoring of the capacities underlying making and experiencing art, the experimental phenomenology is a candidate to provide the criteria for integration of different approaches and evidence. Many results of experimental phenomenology are of course significant parts of the Gestalt psychology legacy. However, the experimental phenomenology is not committed to the principle that artworks are “pure” instances of the law of self-organization, which led Arnheim [40] to refuse the artistic value of abstract expressionism. Metzger’s classification of perceptual properties into structural, global, and expressive qualities, Katz’ research into color and tactile modes of appearance account in a neutral way for the capacity of selecting perceptual chunks of the environment, turning them into the representational features and the mode of appearance of composition [41,42,43].

## 6. Conclusions

The claims Arnheim made about the state and the prospects of the psychology of art can be reduced to a warning against the tendency to cling to conventional concepts of art or to derive the constructs and the variables of the psychology of art from general principles instead of addressing the questions that arise from the variability of the phenomena of art’s production and experience. If art is treated as a legitimate source of variability, the psychology of art is the study of psychological factors that capture that variability, namely the connection of capacities and the specialization of perceptual modules for art’s production and experience. The domain of the psychology of art partially overlaps with other sciences, but that does not mean that the research is parasitic or that teaching psychology of art is compiling a syllabus of alternative theories whose validity cannot be decided by any independent evidence. 

The research can be carried out autonomously according to the standard methods of science while preserving the integration with other sciences and disciplines, as it is the case within the Cognitive Sciences framework. Future research has fundamental issues to address. What is the model that specifies the specialization function? What is the architecture that allows the recruitment of perceptual abilities and the connection of cognitive capacities? Composition can be described as a mapping. What kind of correspondence is it? Is it a one to many correspondence? Does it hold from any (piece of) perceptual regularity to any (part of) composition? Composition is related to the alteration of medium. Does it rely only on the invariance of perception to assure recognizability? It seems implausible, because the alteration involves solving the problems of representation given the medium and the choice of the technique, which accounts for style. To which extent is the variation admitted? Early reactions to fauvist or cubist paintings suggest that the range of admitted variation, which can characterize the style just because it is not attributed to the represented subject, might not be so small. What about recognition adaptation then? 

The prospects for teaching psychology of art do not look as dismal as it might seem if one considered Arnheim’s puzzle unsolvable. On the contrary, the research that address questions specific to the subject matter of the discipline will provide phenomena and findings, and will set a standard for the evaluation of contrasting evidence and competing theories.
